# Full-Genome Sequence of a Hepatitis B Virus Genotype F1b Clone from a Chronically Infected Chilean Patient

**DOI:** 10.1128/genomeA.01075-14

**Published:** 2014-10-23

**Authors:** Sergio Hernández, Mauricio Venegas, Javier Brahm, Rodrigo A. Villanueva

**Affiliations:** a Laboratorio de Virus Hepatitis, Facultad de Ciencias Biológicas, Universidad Andrés Bello, Santiago, Chile; b Sección de Gastroenterología, Hospital Clínico Universidad de Chile, Santiago, Chile

## Abstract

The hepatitis B virus (HBV) is a DNA virus belonging to the *Hepadnaviridae* family. Viral isolates have been classified into 10 genotypes, named from A to J, and several subtypes. We report the full-genome sequence from a single molecular clone of HBV genotype F1b, amplified from a chronically infected Chilean patient.

## GENOME ANNOUNCEMENT

Hepatitis B virus (HBV) chronic infections are a major risk for patients with cirrhosis and hepatocellular carcinoma. The HBV genome is a partially double-stranded 3.2-kb DNA that replicates through reverse transcription of an RNA intermediate, globally introducing variability to the viral spread. HBV isolates are classified into 10 genotypes (named A–J) and several subtypes, with a differential geographic distribution (1, 2). In general, genotypes B and C are prevalent within Asia, whereas genotypes A and D circulate in Europe and the United States (1). Genotype G displays a global distribution, and genotype H circulates in Central America (3, 4). Genotypes I and J have been reported in regions in Asia (5, 6). Finally, HBV genotypes E and F are prevalent in Africa and Central and South America (7). Importantly, the genetic diversity, pathology progression, and antiviral therapy response (8, 9) of the European, North American, and Asian genotypes (A–D) (10, 11) have received the greatest attention and more pharmaceutical efforts than those prevalent in other regions (E-H) (12, 13). Similarly, virological and clinical properties of HBV genotypes have been only systematically studied for genotypes A–D. In broad terms, genotype B is more related to mild liver diseases than genotype C, and genotype D has a less positive prognosis than genotype A (14). Other genotypes, including genotype F, have not been thoroughly studied at the clinical or molecular levels.

In previous studies, the HBV genotype F was found to be the most prevalent in Chile (84%) (15), and phylogenetic analyses of complete genome sequences from crude amplified viral DNA were found to correspond to subgenotype F1b (16).

Viral DNA extracted from serum (sample HCUCH4) was amplified as previously described (16, 17), and then ligated with a TOPO XL PCR vector and transformed into TOP10 *E. coli* (Life Technologies). Screening was based on white/blue selection with X-gal. Plasmid DNA was subjected to restriction mapping and fragment size estimation. One clone was completely sequenced from both strands of the viral DNA (Macrogen Inc.), using primers spaced by approximately 500 nucleotides. Consensus sequences were obtained by alignment of both sequenced strands using MegAlign software from the DNAStar package (LaserGene, Inc.). Assembled viral sequences gave 3,215 nucleotides, with canonical HBV overlapping open reading frames for PreC (HBe, 639 nt), C (HBc, 552 nt), X (HBx, 465 nt), PreS1 (LHBs, 1203 nt), PreS2 (MHBs, 846 nt), S (SHBs, 681 nt), and P (Pol, 2532 nt). This isolate was named clone HBV 4.5. Its DNA sequence was compared to that of sample HCUCH4 (GenBank accession number HM585186), resulting in 99% genetic identity, with the changes in clone HBV 4.5 with respect to HCUCH4: A149G, A1149G, and G1698A. Since digestion of the vector release a circularizing HBV genome with no additional sequences, its availability will be important for the initiation of molecular studies on one of the lesser-studied HBV genotypes, such as genotype F, prevalent not only in Chile but also in Central and South America.

### Nucleotide sequence accession number.

The sequence is available in GenBank under accession number KM233681.
